# Supplementary material for: Scalable Production of a De Novo SARS-CoV-2 Antiviral Miniprotein in Escherichia coli
Source: Pharmaceutics. 2026 Sep 3;18(9):1110. doi: 10.3390/pharmaceutics18091110 (PMC13610847; doi:10.3390/pharmaceutics18091110)
Supplement: Supplementary file 1 [file pharmaceutics-18-01110-s001.zip › pharmaceutics-4405511-supplementary.pdf]

Supplementary Material

Description of Analytical Methods utilized in the study

a. Visual Appearance: The visual appearance of the drug substance is assessed under controlled lighting conditions with standardized backgrounds to evaluate color and clarity. Color is compared with the Yellow Reference Solution prepared according to Ph. Eur. <2.2.2>, and clarity is evaluated using the turbidity reference solution prepared as described in Ph. Eur. <2.2.1>. The sample is placed in a clean, transparent container and gently agitated to ensure uniformity. This test is performed during stability studies to monitor potential changes in appearance.

b. pH: The pH of the drug substance is determined potentiometrically using a calibrated pH meter equipped with a glass indicator electrode and a suitable reference electrode, in accordance with USP <791>. Samples are equilibrated to 25 ± 2 °C before measurement. Calibration is performed prior to use with three NIST-traceable buffers at pH 4.01, 7.00, and 10.01, and the electrode slope is verified to be within 90% to 105%. The electrode is rinsed with purified water and blotted dry before immersion, and the sample is stirred gently to prevent air bubble formation. System suitability is confirmed when the pH 7.00 buffer offset is within ±30 mV of its certified value. This test is used in stability studies to detect pH shifts.

c. Viscosity: Viscosity is measured using a calibrated rotational viscometer at 25.0 ± 0.5 °C, following USP <911>. The formulation includes 0.1% hydroxypropyl methylcellulose (HPMC) to adjust viscosity and improve solution stability. The appropriate spindle for the expected viscosity range is immersed in the sample, with care taken to avoid air bubbles. Torque is recorded once a stable reading is achieved. This method is used in stability studies to ensure rheological properties are maintained.

d. Protein Concentration:is determined using slope spectroscopy with a UV spectrophotometer equipped with a variable-path-length fiber. The method relies on the linear relationship between absorbance at 280 nm and path length at a constant protein concentration. Absorbance is measured at ten path lengths, and the slope is converted to mg/mL using an extinction coefficient of 0.752. Scatter correction is performed using absorbance readings at 320 nm and 350 nm. Samples are equilibrated to 20 ± 5 °C before loading into the cuvette, and the software calculates the concentration. System suitability is verified using a bovine serum albumin (BSA) reference standard within ±5% of the target concentration, as confirmed by linear regression analysis. This method is used in stability studies to monitor protein content over time and has been qualified for its intended analytical use.

e. Binding Affinity by Octet BLI: Binding affinity is measured using label-free Bio-Layer Interferometry (BLI), which detects nanometer-scale changes in optical interference as IPD-52520 binds to the SARS-CoV-2 Spike Receptor Binding Domain (RBD) immobilized on High Precision Streptavidin (HPS) biosensors. The assay includes an initial baseline measurement, ligand loading, baseline re-equilibration, analyte association at multiple concentrations (1.111-30 nM), and dissociation for 1,800 seconds at 30 °C with orbital shaking at 1,000 rpm. Reference subtraction is performed, and kinetic data are globally fitted to a 1:1 Langmuir model to determine the association rate constant (ka), dissociation rate constant (kd), and equilibrium dissociation constant

(KD). This method is used in stability studies to monitor changes in binding activity over time and has been qualified for its intended analytical purpose.

f. Purity by SEC-HPLC: Purity is determined by size-exclusion chromatography using high-performance liquid chromatography (SEC-HPLC) to assess the presence of high- and low-molecular-weight species. The analysis is performed on a TSKgel® G3000SWXL column (7.8 mm × 30 cm, Tosoh Biosciences) with a mobile phase composed of 2.7 M sodium chloride, 54 mM potassium chloride, 86 mM sodium phosphate, and 28 mM potassium phosphate, adjusted to pH 7.2. Samples are diluted to 1 mg/mL with the mobile phase and detected at 280 nm.

g. The trimer peak generally elutes around 8.0 minutes, with higher-molecular-weight species eluting earlier and lower-molecular-weight species eluting later. Size variants are quantified by integrating peaks within predefined boundaries for high-molecular-weight, main, and low-molecular-weight species. System suitability is confirmed by injection precision. The method is used in stability studies to monitor purity changes over time and has been qualified for its intended analytical application.

h. Purity by CE-SDS: Purity is also assessed using capillary electrophoresis-sodium dodecyl sulfate (CE-SDS) under non-reducing conditions, which is suitable for IPD-52520 since it lacks disulfide bonds. Samples are combined with an SDS-containing denaturant, heated at 75 °C for 10 minutes, then cooled to ambient temperature before hydrodynamic injection into a fused-silica capillary (50 μm ID, total length 30.2 cm, effective length 20 cm) equilibrated with separation buffer. Electrophoresis is performed at 15.0 kV and 20 °C with UV detection at 214 nm.

i. Peaks corresponding to the monomer (16.6 kDa), fragments, and high-molecular-weight species are integrated, and purity is expressed as the percentage of the monomer peak area relative to the total peak area. System suitability is verified by migration consistency. The method is used in stability studies to monitor changes in purity over time and has been qualified for its intended analytical purpose.

j. Purity by AEX-HPLC: Charge variant distribution is determined by anion-exchange high-performance liquid chromatography (AEX-HPLC) using a BioSuite DEAE column (10 μm, 7.5 × 75 mm, Waters). The column is equilibrated with a low-ionic-strength buffer containing 20 mM sodium phosphate and 0.5 mM EDTA at pH 6.3. Elution is carried out with a programmed salt gradient using 1 M NaCl at a flow rate of 0.5 mL/min and 25 °C. Detection is performed by fluorescence with 280 nm excitation and 350 nm emission.

k. The major peak, representing a species with an isoelectric point (pI) of 5.3, elutes at approximately 18.0 minutes, with basic variants eluting earlier and acidic variants later. Quantification of charge variants is achieved by integrating peaks within predefined boundaries for basic, main, and acidic species. System suitability is confirmed by injection precision. This method is used in stability studies to monitor charge heterogeneity over time and has been qualified for its intended analytical use.

l. Identity by SEC-HPLC: The identity of the drug substance is verified using size-exclusion chromatography with high-performance liquid chromatography (SEC-HPLC), which separates analytes by hydrodynamic volume. The column is packed with a porous silica-based stationary phase, specifically a TSKgel® G3000SWXL column

(7.8 mm × 30 cm, Tosoh Biosciences), equilibrated with an aqueous buffer containing 2.7 M sodium chloride, 54 mM potassium chloride, 86 mM sodium phosphate, and 28 mM potassium phosphate, adjusted to pH 7.2. The method confirms that the sample's molecular weight matches that of the reference standard, which has been characterized with size markers, specifically the trimer form. Identity is demonstrated by comparing the retention time of the sample's major peak with that of the reference standard, using UV detection at 280 nm and a flow rate of 1 mL/min at 25 °C. System suitability is confirmed through injection precision and matrix interference assessments to ensure accurate analyte identification. The method has been validated for use in identity confirmation.

m. Identity by CE-SDS: The identity of the drug substance is further confirmed by capillary electrophoresis-sodium dodecyl sulfate (CE-SDS) under non-reducing conditions by comparing the sample's migration profile with that of a reference standard characterized for molecular weight using size markers. Electrophoresis is performed at 15.0 kV and 20 °C with UV detection at 214 nm. Concordance in migration time between the sample and the reference standard is used to verify identity. System suitability is confirmed based on migration consistency, and the method has been qualified for use in identity confirmation.

n. Residual Host Cell DNA (HCD): Residual host cell DNA (HCD) is quantified by quantitative polymerase chain reaction (qPCR) in accordance with USP <509>, using the PrepSEQ™ Residual DNA Sample Preparation Kit (Applied Biosystems, 4413686) and the resDNASEQ™ Quantitative E. coli DNA kit (Applied Biosystems, 4458435). DNA extracted from the sample, calibration standards (ranging from 0.03 to 300 pg/μL), and a no-template control (NTC) are amplified in parallel. Residual HCD content is calculated from cycle threshold (Ct) values and converted to ng per 20 mg of drug substance, based on protein concentration determined by slope spectroscopy. System suitability is demonstrated by the NTC Ct value and the linearity of the calibration curve, typically assessed by the coefficient of determination (R^2). The method has been qualified for its intended analytical use.

o. Residual Host Cell Protein (HCP): Residual HCP is measured using a qualified sandwich enzyme-linked immunosorbent assay (ELISA) kit specific to E. coli (Cygnus, F1020), following USP <1132>. Samples are diluted at least 1:32,000, and a four-parameter logistic (4PL) calibration curve spanning 3 to 200 ng/mL is used. Absorbance is measured at 450 nm with a reference at 650 nm, and concentrations are converted to ng per μg of drug substance and normalized to the protein concentration determined by slope spectroscopy. System suitability is confirmed by the linearity of the calibration curve, typically evaluated by the coefficient of determination (R^2), and further verified by spike-recovery, with acceptable recovery ranging from 70 to 130%. The method has been appropriately qualified for its intended analytical purpose.

p. Bacterial Endotoxin (BET): Bacterial endotoxin levels are measured using the kinetic turbidimetric Limulus Amebocyte Lysate (LAL) assay in accordance with USP <85>. Samples are diluted to ensure testing within the Maximum Valid Dilution (MVD). Each test includes negative and positive product controls, as well as a standard endotoxin series (ranging from 0.01 to 100 EU/mL) to verify assay validity. The increase in turbidity is monitored kinetically, and endotoxin concentration is calculated by

comparing it with the calibration curve. Results are expressed as Endotoxin Units (EU) per milligram of drug substance, based on protein concentration determined by slope spectroscopy.

q. Bioburden: Microbial bioburden is evaluated according to USP <61> by membrane filtration. Samples are filtered through a sterile 0.45 μm membrane filter, rinsed, and then incubated for bacterial enumeration on Soybean-Casein Digest Agar (SCDA) at 30 to 35 °C for 3 to 5 days, and for fungal enumeration on Sabouraud Dextrose Agar (SDA) at 20 to 25 °C for 5 to 7 days. Colony-forming units (CFU) are reported as CFU/mL. The suitability of the culture media is confirmed by growth promotion tests.

r. Residual Kanamycin: Residual kanamycin levels are determined by reversed-phase high-performance liquid chromatography (RP-HPLC) on an XBridge BEH C18 column (5 μm, 4.6 × 250 mm, Waters), equilibrated with 40% acetonitrile. Chromatographic separation is performed at 37°C and a flow rate of 0.75 mL/min, with UV detection at 240 nm. Before analysis, samples, calibration standards (0.5 to 50 ppm), and spiked samples are derivatized with triethylamine and phenyl isocyanate at 70°C for 10 minutes. System suitability is confirmed by injection precision and the linearity of the calibration curve, typically indicated by the coefficient of determination (R^2), and is further supported by spike recovery, which should range from 80% to 120%. The method has been validated for its intended analytical purpose.

s. Residual IPTG: Residual isopropyl β-D-1-thiogalactopyranoside (IPTG) is quantified by ion-exclusion high-performance liquid chromatography (IE-PLC) using a Coregel ION 300 column (7.8 × 300 mm, Transgenomic) equilibrated with 2.5 mM sulfuric acid. Chromatographic separation is performed at 40 °C and a flow rate of 0.4 mL/min, with UV detection at 210 nm. Before analysis, samples are diluted threefold with phosphate-buffered saline (PBS), and calibration standards are prepared at concentrations of 0.2 to 1.6 μg/mL. System suitability is confirmed by injection precision and the linearity of the calibration curve, typically demonstrated by the coefficient of determination (R^2), and further supported by spike recovery, with acceptable recovery ranging from 70% to 130%. The method has been validated for its intended analytical purpose.

t. Residual PEI: Residual polyethylenimine (PEI) is measured by reversed-phase high-performance liquid chromatography (RP-HPLC) with a charged aerosol detector (CAD) and a Symmetry C4 column (5 μm, 4.6 × 150 mm, Waters). Chromatographic separation uses a programmed gradient of 0.1% trifluoroacetic acid (TFA) in acetonitrile/isopropanol (50/50, v/v) at a flow rate of 1 mL/min and 60 °C. Samples are prepared by precipitating proteins with ammonium sulfate, and calibration standards are prepared at concentrations of 0.003 to 0.03%. System suitability is verified by injection precision and the linearity of the calibration curve, typically expressed by the coefficient of determination (R^2), and further confirmed by spike recovery, with acceptable recovery between 70 and 130%. The method is qualified for its intended analytical purpose.

u. Residual Tergitol 15-S-9: Residual Tergitol 15-S-9 is determined by mixed-mode high-performance liquid chromatography with a charged aerosol detector (CAD), using an Oasis MAX Online column (30 μm, 2.1 × 20 mm, Waters). Chromatographic separation is performed using a programmed gradient of 0.1 % trifluoroacetic acid (TFA) in methanol at a flow rate of 1 mL/min and 60 °C. System suitability is confirmed by

injection precision and the linearity of the calibration curve, which ranges from 0.01 to 0.2 % and is typically demonstrated by the coefficient of determination (R^2). Spike recovery further supports system suitability, with acceptable recovery ranging from 80 to 120 %. The method has been qualified for its intended analytical use.
